# Creating a safe space for patients and staff members in eye care facilities

**Published:** 2021-07-20

**Authors:** Israel Gnanaraj, Thulasiraj D Ravilla

**Affiliations:** 1 Principal Architect: Design Collaborative, Pondicherry, India.; 2 Executive Director: Lion’s Aravind Institute of Community Ophthalmology (LAICO), Madurai, India. Director-Operations: Aravind Eye Care System, Madurai, India.

**The way hospitals are designed and used has a significant impact on efficiency and patient safety. In this article, we explain how to make buildings safer for patients and staff members.**

**Figure:**
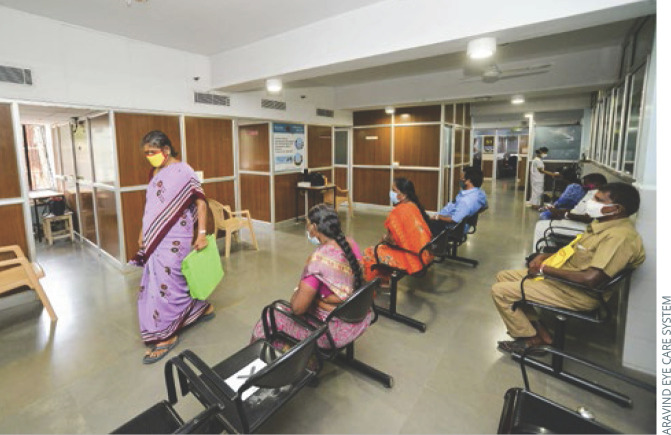
Allocate enough seating for waiting patients and allow for social distancing. **INDIA**

People visit hospitals to get well. However, the potential for falls, accidents, cross-infection and exposure to hazards are high in hospitals due to the inherent complexity of hospital buildings, equipment, and procedures. Eye patients – especially those experiencing visual loss – may find it intimidating to come to a hospital, particularly for the first time.

Good hospital design should be people centric: it should put the needs of patients and staff members first. It should:

- Keep patients physically safe
- Reduce patients’ anxiety
- Provide a comfortable and efficient working environment for staff members.

## Building configuration and workflow

We need to recognise two important factors:

1. In an eye hospital, most patients tend to be visually impaired and elderly.
2. Some patients are likely to be the source of cross-infections due to other illnesses such as COVID-19.^1^

**Case study: Designing a better patient journey**

In a large eye hospital in a South East Asian country, patients had to walk a significant distance from the registration desk to the area where visual acuity was being measured. Patients then had to walk back to registration and continue an equal distance in the opposite direction to be seen by a junior doctor. Next, patients had to walk up a flight of stairs to get to the refraction area, and then up another flight of stairs to speak to the consultant. The laboratories were on yet another floor. With this layout, the hospital seemed congested as patients were walking back and forth, in different directions. The irony was that there was more than adequate space on the entry-level floor to accommodate each of these services.

To improve the situation, the space was reorganised so that all the services were positioned next to each other, in the same order that the patients would be visiting them. As a result, the outpatient flow was a lot more efficient, it took much less time for each patient to complete their visit, and the hospital seemed more spacious. It would have been even more efficient if such optimal flow was planned when the hospital was designed.

The hospital should therefore be designed to avoid overcrowding and make the patient’s journey through the physical space of the hospital as easy and safe as possible (see panel).

Good signage is important as it helps patients to know where they are, and where they need to go next. Some hospitals employ people, or use volunteers, to guide patients through the hospital; this can help to improve the safety of patients who have visual impairment.

It is important to position the various services in the same order that patients would need them (e.g., registration, followed by visual acuity testing, instilling eye drops, slit lamp examination, etc.). This is also known as the ‘workflow requirements’. A good understanding of the workflow requirements in an eye department or hospital, together with an appreciation of patient needs, will help when designing an efficient and safe hospital or making changes to an existing facility.^2^

The physical seating space allocated for people waiting to be seen at each of these stages should match the number of patients expected and include appropriate social distancing to avoid cross-infection. When this isn’t done, it results in bottlenecks, increased travel distances, and back-and-forth movement of patients and staff members. The greater the movement, the greater is the likelihood of cross-infection and accidents. Efficiency will also be lower.

### Planning for growth

As a hospital’s reputation grows, so does the number of patients and staff members. New equipment and procedures also require additional space. In these circumstances, it is important that additional spaces for outpatient services and operating rooms are set up next to, or very near to, those that exist already. This would ensure that building layout continues to mirror the workflow.^2^ Without detailed planning, expansion can involve duplicate equipment, duplicate staffing, and more movement for patients. Insufficient allocation of space may also result in overcrowding and compromise patient safety.

### Friendly design

Safety considerations in hospital buildings can go hand-in-hand with creating a friendly environment and an enjoyable experience for patients.

Courtyards, skylights, corridors leading towards points of visual interest or views, special niches or recesses for parking stretchers and equipment, and the use of colour and texture can serve functional needs and enhance patients’ experience of the space. Natural light, plants, music, and art can also make the hospital environment more enjoyable for patients and staff members.

## Keeping patients physically safe

It is all too easy to accept things as they are and simply work around issues that are potentially unsafe for patients. We therefore recommend carrying out periodic reviews (ideally carried out by an external person) to identify potential hazards and address them proactively.

The following could serve as a a checklist and/or guide.

### Preventing falls, slips and trips

- Guard rails and barriers must be of sufficient height and strength in both interior and exterior spaces.
- Interior and exterior floor surfaces must have sufficient traction to prevent slips and falls.
- Ramps, stairs and corridors should be brightly lit and clearly visible.
- Avoid sudden changes in floor levels. Where this is unavoidable, put up a highly visible warning sign and mark the edges with high contrast tape or painted lines or, ideally, there should be a ramp with hand rails.
- Areas where water is used must have good drainage so that floors remain dry.

### Preventing collisions

- All clear glass partitions must have visual barrier strips or designs to prevent anyone mistakenly trying to walk through them.
- Swing doors are preferable over sliding doors. eople in a hurry tend to assume doors swing open, so they tend to walk into sliding doors. It is also easier to operate swing doors while pushing wheelchairs and trolleys.
- Doors need appropriate door furniture: handles if the door must be pulled, and a flat plate or a horizontal bar when a door has to be pushed open.
- Where two-directional movement is expected, there should be double swing doors with view panels.
- The speed and force of auto-close doors must be calibrated to avoid accidents.
- Avoid projections, such as low hanging sign boards, in areas used by people.
- Doors must not open directly onto stairs or ramps.

**Figure:**
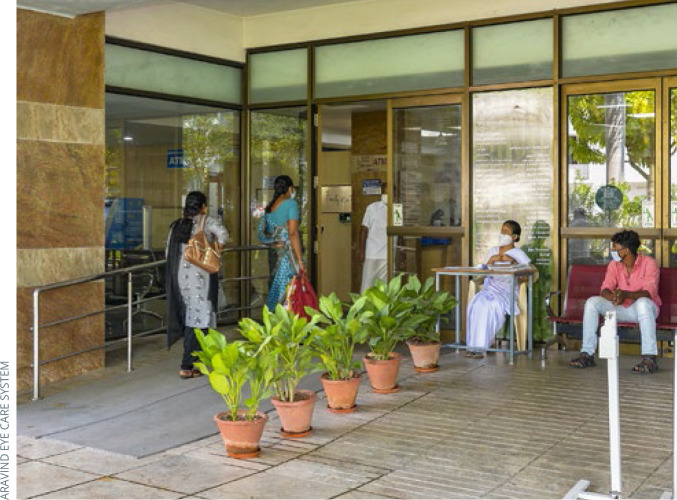
Plants can make the hospital environment more enjoyable for staff members and patients. **INDIA**

### Occupational health and safety

- Task lighting shoud be appropriate for the task being carried out. Avoid glare on computer screens and work surfaces due to bright lights and windows. Adjustable blinds can help in controlling glare from windows. Good lighting ensures that labels and reports can be read properly and reduces the risk of error.
- Keep noise at an acceptable level. A quiet environment enhances concentration and productivity. Communication between patient and provider is clearer, less ambiguous, and more effective. External noise can often be reduced by closing the windows or doors, as appropriate. Fixing air-conditioning or fans can often reduce internal sources of noise. If echoing is an issue, use sound-absorbing materials like fabric or acoustic tiles.
- Position furniture, equipment, and cables to enhance ease of work and safety.
- Use and store all hazardous equipment as recommended. Proper safety warnings must be visible.
- Staff members working with potentially hazardous equipment, like lasers, must have appropriate safety equipment.
- Staff members need access to washrooms/toilets and areas where they can rest or socialise away from patients. This is essential, but often missed.

### Ventilation and indoor air quality

- Maximize natural and cross-ventilation. Courtyards can bring natural ventilation and light into hospital buildings. Courtyards also act as social and spill-over spaces, are a great reference point for navigation, and connect the outdoors with the indoors. Courtyards can also expand capacity during emergencies.
- Where natural ventilation is not feasible, plan to use mechanical ventilation in accordance with local standards and requirements.
- Hospital air conditioning needs are very specific and may not be met by domestic units; it is therefore advisable to consult experts suppliers. It is also important to have optimal relative humidity.
- Use materials and furnishings that are low emitters of indoor air contaminants and volatile organic compounds (VOCs).

### Electrical safety

- All electrical installations should be done in strict accordance with the respective country’s electrical safety codes.
- Good earthing increases safety as well as the life of sensitive electrical equipment.
- Integrate safety devices in the electrical system to prevent short circuits and overload.
- Label all electrical panels. Display circuit diagrams near panels for quick reference during emergencies.
- Use colour coding of switches and power outlets to identify UPS power, power with standby, and power without standby.
- If you notice any damp or wet areas in and around an electrical installation, attend to it as a priority.
- Replace exposed or frayed cables immediately.
- Do not plug multiple equipment into the same outlet. The wiring or the outlet may not support the total power load. As far as possible, avoid the use of multi-plugs, unless it is for low-wattage use.
- While upgrading wiring or while planning new facilities, consider providing 25% more power outlets than the identified need.
- Plug type and voltage provision vary between countries. With imported equipment, check that the voltage is compatible and replace the plug if needed.
- Set up uninterrupted power supplies (UPS) and surge protectors in case of unexpected power surges or power cuts and extreme circumstances such as lightning strikes.

### Hazardous materials and waste handling

Hospital processes may use and or generate hazardous materials.

- Identify possible hazardous materials that need to be handled. Set out clear guidelines or standard operating procedures (SOPs) to isolate, remove, and manage such materials.
- Plan separate holding/storage areas for hazardous materials, away from spaces used by the public.
- The waste generated in hospitals, including contaminated materials, sharps, etc., require careful handling. Put separate, colour-coded waste collection bins in every area where waste is generated, as well as posters to encourage staff members, patients and visitors to separate waste at source. Position the bins so waste can be disposed of easily and the bins themselves can be removed safely.
- Dispose of contaminated waste according to local regulations. In the absence of such regulations, follow the practices advocated by the World Health Organization (see article on p. 12).

## Dangers, disasters, and security threats

Although rare, fires, earthquakes, extreme weather, and violence or security threats can have a devastating impact on an eye service and its staff members and patients. It is vital that buildings are planned with these threats in mind and that periodic mock drills and checks are carried out to ensure that precautionary measures are sufficient.

### Fire protection

- In areas where there are wild fires, maintain an adequate fire-break around the hospital building to prevent the fire spreading to the building. Fire-breaks are kept clear of vegetation (apart from short grass) and can be from 5 to 15 metres wide, depending on the height of the surrounding vegetation and local weather conditions.
- Modern hospital buildings use electromechanical systems and fossil fuels which increase the risk of fire. Fire protection engineers must be involved in all aspects of the hospital building’s design in order to ensure fire protection measures are in place.
- All building and fire regulations or codes must be adhered to.
- Exit paths should be clearly identified and kept clear of obstruction at all times.
- In multi-storey buildings, areas of refuge – a place to safely gather, before being rescued by fire personnel – should be designated and maintained.
- Proper fire detection, alarm, and suppression systems (such as sprinklers) can save lives and property.
- Emergency lighting and power systems should function and be kept in readiness at all times by carrying out regular checks and maintenance.
- It is important to have fire-related information and signs clearly displayed in local languages.
- Regular fire drills and training of staff members should be given priority.

### Earthquakes

- Most national building codes specify the highest degree of earthquake resistance for hospital buildings. Hospitals not only have to be safe for their own patients and staff members, but should also be able to function fully to provide relief to injured people in the aftermath of an earthquake.
- If the hospital is built in an area where earthquakes can occur, experienced structural engineers must check the whether the building is able to withstand them.
- All large and heavy equipment should be properly anchored at all times to ensure that they do not cause damage during earthquakes.
- Emergency preparedness for earthquakes should be part of the safety protocol of hospitals.

### Storms, heavy rain, snow, flooding, landslides, etc.

- Many natural and seasonal occurrences could create safety issues for hospitals.
- Selecting a good site – higher ground, away from flowing water bodies, well drained – will reduce the risk that extreme weather will affect the hospital building.
- One may not have the luxury of an ideal site. Good engineering design could greatly reduce the risk of damage from extreme weather.
- All equipment and systems that are meant to work during such eventualities, like storm drains, pumps, etc., must be kept in good in working condition at all times and located in areas that are unlikely to be affected by flooding and so on.

### Security from human threats

- Security risks could range from pilferage and theft to vandalism and terrorist attacks. Hospitals are open to everyone, and this makes it vulnerable to threats. However, the number of entrances could be planned, balancing functional needs, efficiency, and security concerns.
- Surveillance systems should be planned without compromising privacy.
- The hospital can be designed so that patients and visitors are restricted to specific areas only.
- Quieter areas, such as parking garages and storage areas, should be closely monitored.
